# Noise and Memristance Variation Tolerance of Single Crossbar Architectures for Neuromorphic Image Recognition

**DOI:** 10.3390/mi12060690

**Published:** 2021-06-13

**Authors:** Minh Le, Thi Kim Hang Pham, Son Ngoc Truong

**Affiliations:** 1 Faculty of Electrical and Electronics Engineering, Ho Chi Minh City University of Technology and Education, Ho Chi Minh City 70000, Vietnam; leminh@hcmute.edu.vn; 2 Faculty of Applied Sciences, Ho Chi Minh City University of Technology and Education, Ho Chi Minh City 70000, Vietnam; hangptk@hcmute.edu.vn

**Keywords:** neuromorphic image recognition, Gaussian noise, memristance variation, memristor array, complementary crossbar, twin crossbar, single crossbar

## Abstract

We performed a comparative study on the Gaussian noise and memristance variation tolerance of three crossbar architectures, namely the complementary crossbar architecture, the twin crossbar architecture, and the single crossbar architecture, for neuromorphic image recognition and conducted an experiment to determine the performance of the single crossbar architecture for simple pattern recognition. Ten grayscale images with the size of 32 × 32 pixels were used for testing and comparing the recognition rates of the three architectures. The recognition rates of the three memristor crossbar architectures were compared to each other when the noise level of images was varied from −10 to 4 dB and the percentage of memristance variation was varied from 0% to 40%. The simulation results showed that the single crossbar architecture had the best Gaussian noise input and memristance variation tolerance in terms of recognition rate. At the signal-to-noise ratio of −10 dB, the single crossbar architecture produced a recognition rate of 91%, which was 2% and 87% higher than those of the twin crossbar architecture and the complementary crossbar architecture, respectively. When the memristance variation percentage reached 40%, the single crossbar architecture had a recognition rate as high as 67.8%, which was 1.8% and 9.8% higher than the recognition rates of the twin crossbar architecture and the complementary crossbar architecture, respectively. Finally, we carried out an experiment to determine the performance of the single crossbar architecture with a fabricated 3 × 3 memristor crossbar based on carbon fiber and aluminum film. The experiment proved successful implementation of pattern recognition with the single crossbar architecture.

## 1. Introduction

The memristor, the new fourth basic circuit element, was mathematically proposed by L. O. Chua in 1971 [1] and experimentally demonstrated by the HP lab in 2009 [2]. Since then, memristors have been crucially used to demonstrate neuromorphic computing systems, which were conceptually proposed in 1990 by C. Mead [3]. The nonlinear charge–flux relationship of the memristor, which can be used to simulate the behavior of human synapses [4,5], makes it a promising candidate for neuromorphic systems. Furthermore, the conductance of memristors could be modified and saved by applying programming pulse [4,6], which is the key characteristic of memristors for supporting neuromorphic system implementation.

Interestingly, memristors can be formed as a crossbar array, which is a fully connected mesh of crossing wires [7,8,9]. Two crossing wires in the crossbar are connected by a memristor acting as a switch [7,9]. Memristor crossbars have opened opportunities to implement artificial neural networks on chips where the synaptic weights of network are stored in crossbar array [10,11,12,13]. These potential applications, however, require huge computational tasks and training processes. Recently, other approaches have been proposed where memristor arrays were used for neuromorphic pattern recognition, including speech recognition and image recognition [14,15]. The complementary architecture, in which one memristor crossbar is the inversion of the other, is used for the application of speech recognition [14]. It is based on a logical Exclusive-NOR (XNOR) operation, which measures the similarity of two binary arrays [14]. The twin crossbar architecture employing two identical crossbar arrays has been proven capable of measuring the similarity between an input pattern and the stored patterns as well [15]. The twin crossbar architecture consumes less power than the complementary crossbar architecture for the application of image recognition. In complementary crossbar architecture, the number of ‘1′ bits is always equal to the number of ‘0′ bits, irrespective of the sparsity density of images stored in the crossbars, because the two crossbars are complementary to each other. By contrast, the number of ‘1′ bits in the twin crossbar architecture is dependent on the data density of the images. For this reason, the twin crossbar architecture consumes less power than the complementary crossbar architecture if and only if the images stored in the crossbar array have the number of ‘1′ bits less than the number of ‘0′ bits, for instance, in DCT compressed images [15]. An up-to-date architecture, the single crossbar architecture, obtained by simplifying the Exclusive-NOR operation, needs only one memristor array for implementing the Exclusive-NOR function in pattern recognition tasks [16]. The complementary and twin crossbar architectures accept unipolar inputs, but the single crossbar array accepts bipolar inputs instead. In term of power consumption and area occupation, each type of crossbar architecture has significant advantage as they are applied to the specific application. In particular, the power consumption can be saved in the twin crossbar architecture with DCT compressed images, in which the number of ‘1′ bits is much less than the number of ‘0′ bits [15]. To save area, we can consider the single crossbar architecture, but the unipolar to bipolar circuit must be used in this case [16]. 

All the above crossbar architectures require memristors to operate at a desired memristance value, which is either low resistance state (LRS) or high resistance state (HRS). However, the memristance value varies from device to device due to manufacturing variation or being programmed into an undesired state [17,18,19,20,21]. Memristance variation is one of the factors that degrade the performance of the memristor crossbar circuit [17,18,19]. All of the above crossbar architectures have been tested with clean images. However, the recognition rate of these crossbar architectures may be reduced with noisy images. In this work, we performed a comparative study on the Gaussian noise and memristance variation tolerance of the complementary crossbar architecture, the twin crossbar architecture, and the single crossbar architecture. Based on the results, we determined that the single crossbar architecture produced the best recognition rate among the three architectures for image recognition under the effect of Gaussian noise and memristance variation. We also performed an experiment on the single crossbar architecture with fabricated 3×3 memristor crossbar based on carbon fiber and aluminum film for storing and recognizing three simple patterns.

## 2. Memristor Crossbar Architectures for Neuromorphic Image Recognition

### 2.1. The Complementary Memristor Crossbar

**Figure 1:**
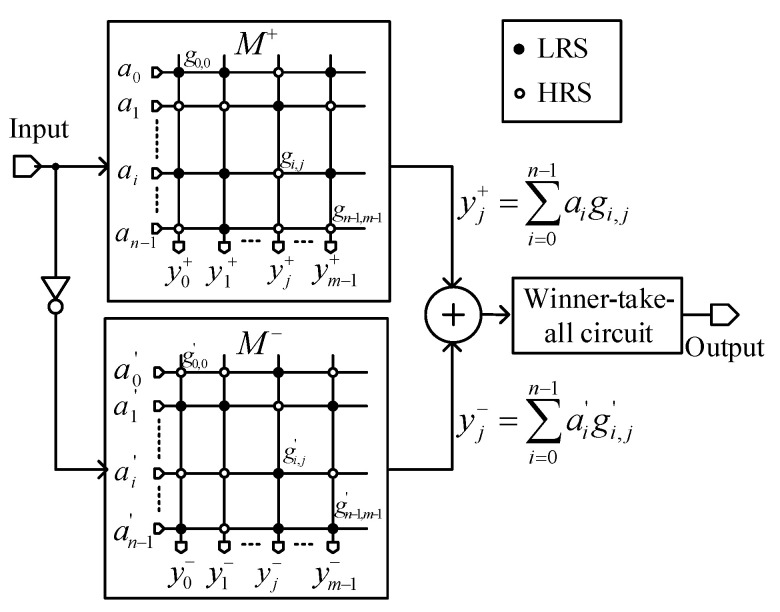
The complementary memristor crossbar architecture (Reproduced with permission from [14], published by SpringerOpen).

A complementary crossbar architecture consisting of two complementary crossbar arrays for pattern recognition is depicted in Figure 1. Here, M+ and M− represent the memristor crossbar and its inversion, which consists of inverted elements of M+, respectively.

In Figure 1, M+ is an array of memristors, which has the size of n × m. At the intersection of the $i^{th}$ row and the $j^{th}$ column, there is a memristor with the conductance of $g_{ij}$ that can be either low resistance state (LRS) or high resistance state (HRS). The LRS and HRS in Figure 1 are shown as a solid black circle and an open circle, respectively. In Figure 1, $g_{00}$ is the memristor conductance at the intersection of the first row and the first column with the value of LRS. The M− consists of the inversed elements of M+, namely the conductance ${g\prime }_{ij}$ at the intersection of the $i^{th}$ row and the $j^{th}$ column in M− is the inversion of $g_{ij}$ in the M+ array. The M+ and M− arrays can be written as matrices as follows:

(1) $$M+=\left[\begin{array}{cccc} g_{0,0} & g_{0,1} & \ldots & g_{0,\left(m-1\right)}\\ g_{1,0} & g_{1,1} & \ldots & g_{1,\left(m-1\right)}\\ \vdots & \vdots & \vdots & \vdots \\ g_{\left(n-1\right),0} & g_{\left(n-1\right),1} & \ldots & g_{\left(n-1\right),\left(m-1\right)} \end{array}\right]\;M-=\left[\begin{array}{cccc} {g^{\prime }}_{0,0} & {g^{\prime }}_{0,1} & \ldots & {g^{\prime }}_{0,\left(m-1\right)}\\ {g^{\prime }}_{1,0} & {g^{\prime }}_{1,1} & \ldots & {g^{\prime }}_{1,\left(m-1\right)}\\ \vdots & \vdots & \vdots & \vdots \\ {g^{\prime }}_{\left(n-1\right),0} & {g^{\prime }}_{\left(n-1\right),1} & \ldots & {g^{\prime }}_{\left(n-1\right),\left(m-1\right)} \end{array}\right]$$

The input pattern that needs to be recognized is a vector with the size of 1 × n. In Figure 1, the input vector that is applied to the M+ array is $A=\left[\begin{array}{cccc} a_{0} & a_{1} & \ldots & a_{n-1} \end{array}\right]$, and its inversion, $A\prime =\left[\begin{array}{cccc} {a\prime }_{0} & {a\prime }_{1} & \ldots & {a\prime }_{n-1} \end{array}\right]$, is applied to the M− array.

To recognize the input vector A, A is applied to the M+ array and A′ is applied to the M− array to implement the XNOR function between A and M:

(2) $$Y=\bar{A⊕M}=AM+A^{\prime }M^{\prime }=A\cdot \left(M+\right)+A^{\prime }\cdot \left(M-\right)$$

In Equation (2), M+ contains prestored patterns of all input vectors that will be recognized. The pattern for recognizing the $j^{th}$ input vector, i.e., the $j^{th}$ input image, is stored in the $j^{th}$ column of M+. All values in M− are the inverted values of the M+ array. The XNOR function is utilized to measure the similarity between the input pattern and the stored patterns. The output vector Y, $Y=\left[\begin{array}{cccc} y_{0} & y_{1} & \cdots & y_{m-1} \end{array}\right]$, contains the similarity scores of the input vector A with the columns of the complementary array [14].

By applying Equation (1) to Equation (2), the output *Y* is calculated as follows:

(3) $$\begin{array}{c} Y=\left[\begin{array}{cccc} a_{0} & a_{1} & \ldots & a_{\left(n-1\right)} \end{array}\right]\cdot \left[\begin{array}{cccc} g_{0,0} & g_{0,1} & \ldots & g_{0,\left(m-1\right)}\\ g_{1,0} & g_{1,1} & \ldots & g_{1,\left(m-1\right)}\\ \vdots & \vdots & \vdots & \vdots \\ g_{\left(n-1\right),0} & g_{\left(n-1\right),1} & \ldots & g_{\left(n-1\right),\left(m-1\right)} \end{array}\right]\\ +\left[\begin{array}{cccc} {a^{\prime }}_{0} & {a^{\prime }}_{1} & \ldots & {a^{\prime }}_{\left(n-1\right)} \end{array}\right]\cdot [\begin{array}{cccc} {g^{\prime }}_{0,0} & {g^{\prime }}_{0,1} & \ldots & {g^{\prime }}_{0,\left(m-1\right)}\\ {g^{\prime }}_{1,0} & {g^{\prime }}_{1,1} & \ldots & {g^{\prime }}_{1,\left(m-1\right)}\\ \vdots & \vdots & \vdots & \vdots \\ {g^{\prime }}_{\left(n-1\right),0} & {g^{\prime }}_{\left(n-1\right),1} & \ldots & {g^{\prime }}_{\left(n-1\right),\left(m-1\right)} \end{array}]=[\begin{array}{cccc} y_{0} & y_{1} & \cdots & y_{m-1} \end{array}]\\ where\;y_{j}=y_{j}^{+}+y_{j}^{-}=\overset{n-1}{\underset{i=0}{\sum }}(a_{i}g_{i,j}+a\prime_{i}g\prime_{i,j}) \end{array}$$

Here, $a_{i}$ is the input voltage representing the input value of either 0 or 1, and $g_{i,j}$ is the conductance of the $i^{th}$ memristor of the $j^{th}$ column. Therefore, $y_{j\;}$ is the $j^{th}$ column-line current representing the similarity between the input pattern and the pattern stored in the $j^{th}$ column. For example, if the input pattern represented by vector A, $A=\left[\begin{array}{cccc} a_{0} & a_{1} & \ldots & a_{n-1} \end{array}\right]$, matches with the pattern stored in the $j^{th}$ column of the array, the column current $y_{j}$ has the largest value in comparison with the other column currents. All column-line currents from $y_{0}$ to $y_{m-1}$ are compared each other in the winner-takes-all circuit, and the largest column current $y_{j}$ is chosen, indicating that the pattern stored in the $j^{th}$ column is the best match with the input pattern.

### 2.2. The Twin Memristor Crossbar

**Figure 2:**
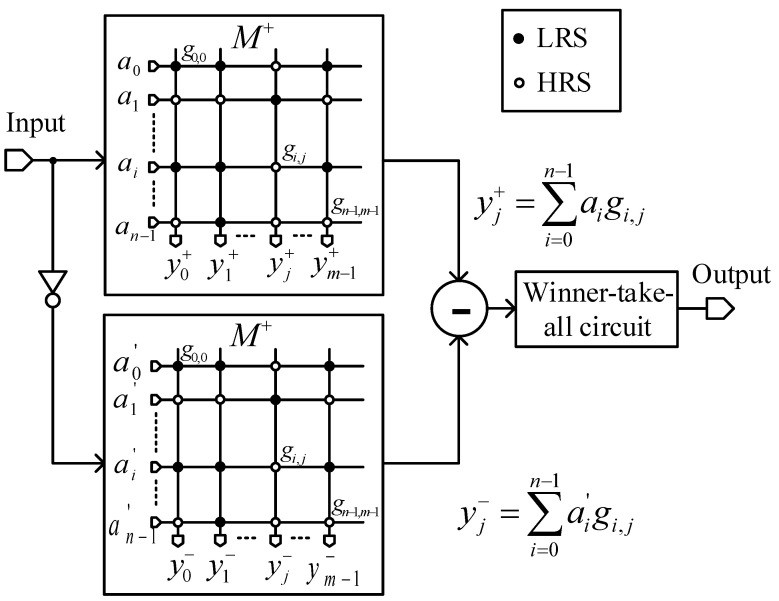
The twin crossbar architecture, which employs two identical crossbar arrays for image recognition (Reproduced with permission from [15], published by IEEE).

The twin crossbar architecture, which employs two identical M+ arrays, has been proven to have the same functionality of the complementary crossbar architecture for image recognition [15]. The architecture of the twin crossbar is conceptually shown in Figure 2.

In this architecture, the XNOR function in Equation (2) is re-expressed as follows [15]:

(4) $$Y=\bar{A⊕M}=AM+A^{\prime }M^{\prime }=AM+A^{\prime }\left(1-M\right)\;=AM-A^{\prime }M+A\prime \;\;\;\;\;\;$$

The A′ in Equation (4) is a constant and has no interaction with array M. Therefore, it can be dismissed when implementing the XNOR function with no effect on the results. Equation (4) can be rewritten as follows [14,15]:

(5) $$Y=\bar{A⊕M}=A\cdot \left(M+\right)-A^{\prime }\cdot \left(M+\right)\;=\left[\begin{array}{cccc} y_{0} & y_{1} & \cdots & y_{m-1} \end{array}\right]\;where\;y_{j}=y_{j}^{+}-y_{j}^{-}=\overset{n-1}{\underset{i=0}{\sum }}\left(a_{i}g_{i,j}-a\prime_{i}g_{i,j}\right)$$

As shown in Equation (5), the twin crossbar uses two identical crossbar arrays for storing patterns instead of two complementary crossbar arrays [15]. The output vector Y is then applied to the winner-takes-all circuit for determining the $j^{th}$ column corresponding to the largest $y_{j}$ that is the best match with the input vector A.

### 2.3. The Single Memristor Crossbar Array

By simplifying the XNOR in Equation (4), a new single memristor crossbar array has been shown to be capable of measuring the similarity between two vectors for the application of image recognition [16]. The XNOR function in Equation (4) is simplified as Equation (6) [16]:

(6) $$Y=\bar{A⊕M}=AM+A^{\prime }M^{\prime }\;=AM+A\prime \left(1-M\right)\;=AM-A^{\prime }M+A^{\prime }\;=\left(A-A^{\prime }\right)M+A^{\prime }$$

In Equation (6), A′ can be dismissed as it has no interaction with the M array. For performing the XNOR function, Equation (6) can be rewritten as Equation (7) [16]:

(7) $$Y=\bar{A⊕M}=IM\;where\;I=\left(A-A^{\prime }\right)$$

In Equation (7), I is a 1×n vector, $I=\left[\begin{array}{cccc} i_{0} & i_{1} & \cdots & i_{\left(n-1\right)} \end{array}\right]$, that is composed of bipolar inputs. For example, if the vector A is A =[101], the vector I will be I =[1−1 1]. Equation (7) can be represented as follows:

(8) $$Y=\left[\begin{array}{cccc} i_{0} & i_{1} & \ldots & i_{\left(n-1\right)} \end{array}\right]\cdot \left[\begin{array}{cccc} g_{0,0} & g_{0,1} & \ldots & g_{0,\left(m-1\right)}\\ g_{1,0} & g_{1,1} & \ldots & g_{1,\left(m-1\right)}\\ \vdots & \vdots & \vdots & \vdots \\ g_{\left(n-1\right),0} & g_{\left(n-1\right),1} & \ldots & g_{\left(n-1\right),\left(m-1\right)} \end{array}\right]\;=\left[\begin{array}{cccc} y_{0} & y_{1} & \cdots & y_{m-1} \end{array}\right]\;where\;y_{j}=\overset{n-1}{\underset{k=0}{\sum }}i_{k}g_{k,j}$$

**Figure 3:**
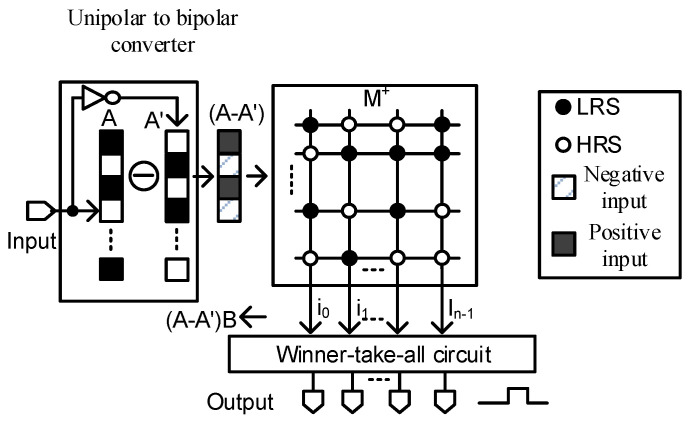
The single crossbar architecture for image recognition (Reproduced with permission from [16], published by IEEE).

Equations (7) and (8) show that this single crossbar architecture uses only one crossbar array to which the bipolarized input vector is applied for pattern recognition. This single crossbar architecture is represented in Figure 3 [16]:

The output vector Y of the XNOR function is then applied to the winner-takes-all circuit to find the largest value $y_{j}$, which means that the $j^{th}$ column of the array matches the input vector A.

## 3. Simulations and Results

**Figure 4:**
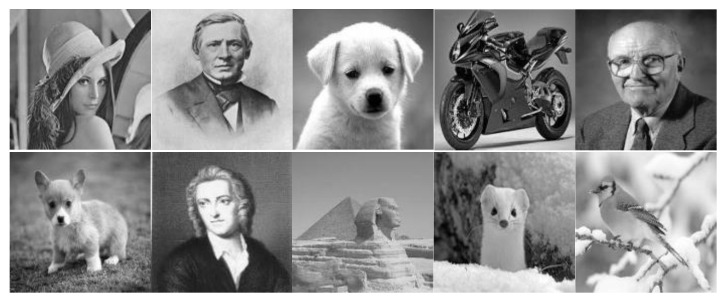
The 10 grayscale images used for testing.

In this work, we first performed a comparative study on noise tolerance of the different crossbar architectures, namely the complementary crossbar, the twin crossbar, and the single crossbar. The 10 grayscale images shown in Figure 4 were utilized for testing. The testing images had the size of 32×32.

**Figure 5:**
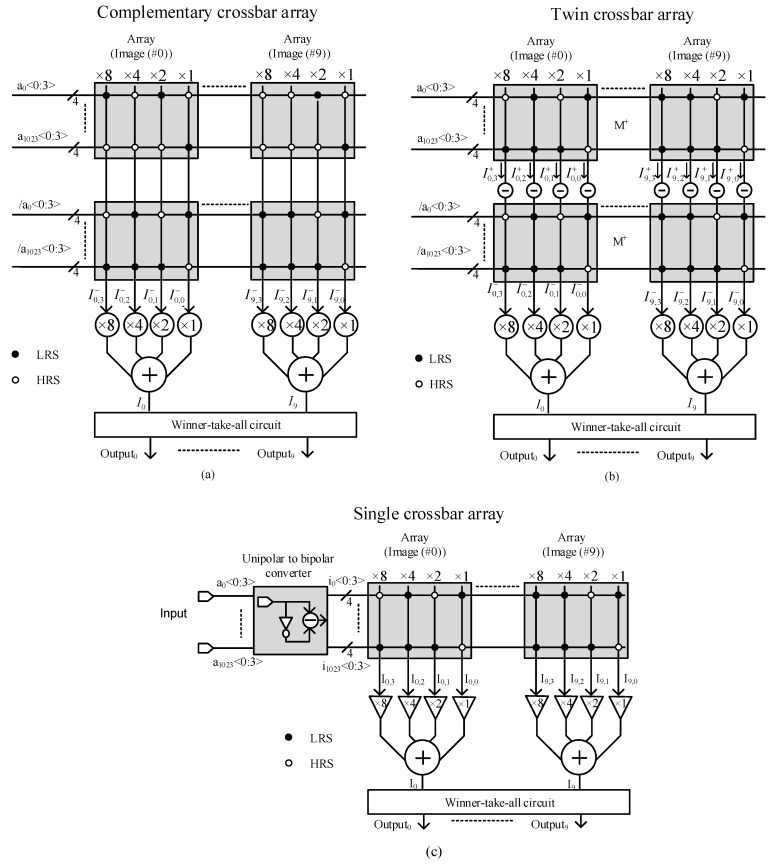
The block diagrams of complementary crossbar architecture (**a**), twin crossbar architecture (**b**) (Reproduced with permission from [15], published by IEEE), and single crossbar architecture (**c**) (Reproduced with permission from [16], published by IEEE) for recognizing 10 grayscale images with the size of 32×32 pixels.

Each image was first converted from the size of 32×32 pixels to a vector with the size of 1×1024 pixels. Each pixel was then digitized by 4 bits [14,15]. Each 4 bit pixel $a_{i}\langle 0:3\rangle$ of one image was stored to four cross-points in four columns that had the weights of 8, 4, 2, and 1 for output calculating. All 10 images were stored to 10 groups with four columns each in the arrays (M+, M−) as patterns for recognizing an input vector. The block diagrams of the crossbar architectures for recognizing images are shown in Figure 5.

For being recognized, each input image was converted to the vector A with the size of 1×1024 pixels. Each pixel in the vector A was then digitized by 4 bits. In the complementary crossbar architecture, the input vector A was applied to the M+ array and A′ was applied to the M− array to perform the XNOR function as shown in Equation (3). In the twin crossbar architecture, the input vector A was fed to the M+ array and A′ was fed to another M+ array for the XNOR function as in Equation (5). In the single crossbar architecture, the input vector A was bipolarized by the unipolar to bipolar convertor before applying to one M+ array for XNOR function as discussed in Equation (8). Here, each bit, the 4 bit 〈0:3〉 of a pixel was multiplied by weights of 1, 2, 4, and 8 before going to the summation block. The $k^{th}$ output, $I_{k}$, contained the amount of the similarity between the input vector A and the $k^{th}$ stored pattern. The winner-takes-all circuit was used to finally choose the maximum $I_{k}$, which showed that the input vector A matched the $k^{th}$ prestored pattern, i.e., $k^{th}$ prestored image [14,15,16].

**Figure 6:**
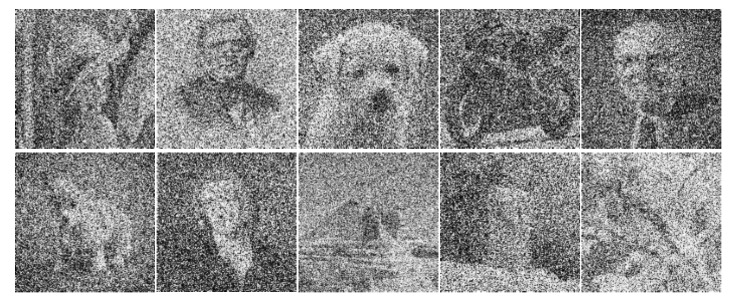
The 10 grayscale images with Gaussian noise at the SNR of −10 dB.

In this study, the three crossbar architectures were tested with input images that had Gaussian noise added. Figure 6 shows the input images after adding Gaussian noise with the signal-to-noise ratio (SNR) of −10 dB.

**Figure 7:**
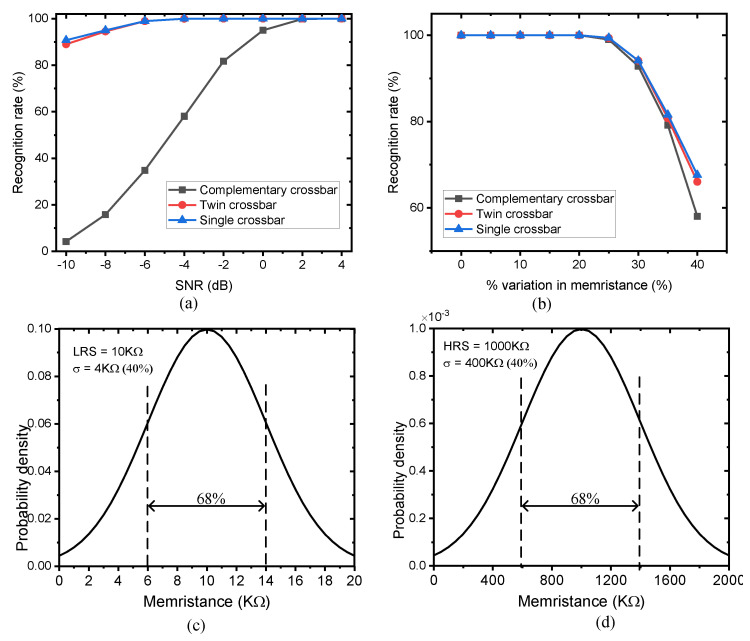
The recognition rates of three architectures: (**a**) images with Gaussian noise added in which the SNR was varied from −10 to 4 dB; (**b**) variation in memristance of arrays; (**c**) statistical distribution of LRS; (**d**) statistical distribution of HRS.

The Gaussian noise was added to the input images with the SNR varied from −10 to 4 dB. The original images were stored in the crossbar array, as conceptually explained above. The images with noise added were then digitized by 4 bits and applied to the complementary crossbar array, the twin crossbar array, and the single crossbar array for recognition. Figure 7a shows the comparison of recognition rates among the three crossbar architectures where the SNR was varied form −10 to 4 dB.

As shown in Figure 7a, the recognition rate of the complementary architecture declined dramatically when the SNR was −10 dB. However, the twin architecture and the single crossbar with bipolar input maintained a recognition rate as high as 89%. In complementary architecture, the column currents are the sum of the column current in the M+ crossbar and the column current in the M− crossbar, as shown in Equation (3). Therefore, the variation of column currents caused by the input noise is increased. In contrast, the twin architecture uses the subtraction in Equation (5), so the current variation caused by the input noise can be compensated. The single crossbar is formulated from the twin architecture, as indicated in Equation (7), so the noise can be slightly compensated at the unipolar to bipolar module. As a result, the single crossbar with bipolar input shows slightly better recognition rate when compared to the twin architecture and complementary architecture. When the SNR was −10 dB, the recognition rate of the complementary architecture, the twin architecture, and the single crossbar with bipolar input were 4%, 89%, and 91%, respectively.

Memristance variation is one of the problems that degrade the performance of memristor crossbar-based applications [17,18,19,20,21]. In this work, we also compared the performance of the complementary architecture, the twin architecture, and the single crossbar with bipolar input with respect to the variation of memristance. In this simulation, the percentage of memristance variation was varied from 0% to 40%. Figure 7b compares the recognition rates of the complementary architecture, the twin architecture, and the single crossbar with bipolar input when the percentage of variation in memristance was increased from 0% to 40%. In the simulation, Gauss distribution was used for memristance variation, as shown in Figure 7c,d. LRS and HRS were assumed to be 10 and 1 MΩ, respectively. As shown in Figure 7c, for LRS, the percentage of variation was 40%, meaning the memristance value varied from (μ − σ) to 14 (μ + σ) kΩ with the probability of 68%. As shown in Figure 7d, for HRS, the percentage of variation was 40%, meaning the memristance value varied from 600 to 1400 kΩ with the probability of 68%.

The twin architecture employs two identical crossbar arrays and is associated with the subtraction in Equation (5), so it can partly compensate the variation of column current caused by the variation in memristance. This explains why twin crossbar showed better recognition rate with variation in memristance as high as 40% when compared to the complementary architecture. The single crossbar with bipolar input had a recognition rate of 67.8%, which was better than the complementary architecture and twin architecture with recognition rates of 58% and 66%, respectively. When the percentage of variation increased higher than 40%, all crossbar architectures would produce very low recognition rate, as implied from Figure 7b. In addition, the column-line currents strongly depend on inputs with LRS memristors rather than HRS memristors; therefore, the variation of LRS memristors degrades the recognition rate more seriously than the variation of HRS memristors.

The statistical simulations showed that the single crossbar array with bipolar input was better than the complementary architecture and the twin architecture for image recognition with input noisy images and variation in memristance. In particularly, for the input noisy images with SNR of −10 dB, the single crossbar showed higher recognition rate by 87% and 2% compared to the complementary architecture and the twin architecture, respectively. Furthermore, the recognition rate of the single crossbar was 9.8% and 1.8% higher than those of the complementary architecture and the twin architecture when the percentage of variation in memristance was as high as 40%.

**Figure 8:**
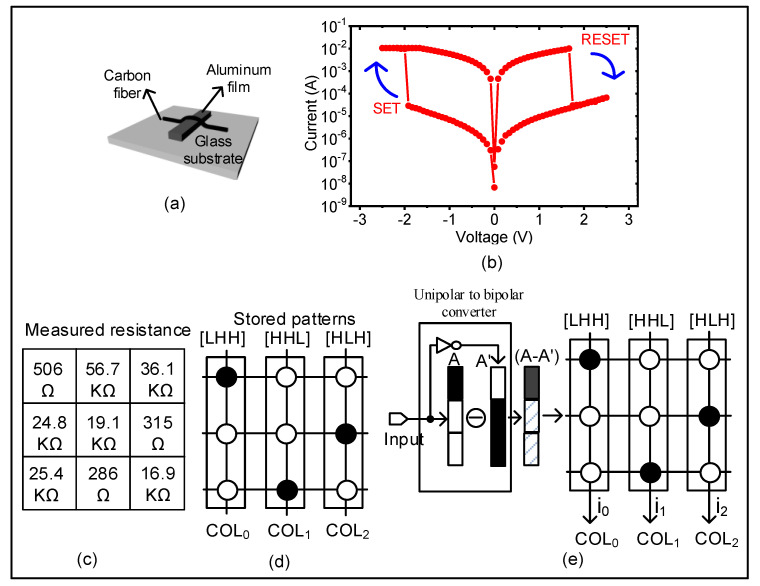
Experimental test of the single crossbar array architecture for recognizing three patterns: (**a**) schematic of the fabricated memristor device based on carbon fiber and aluminum film (Reproduced with permission from [22], published by published by SpringerOpen); (**b**) the measured current–voltage of the fabricated memristor in which the applied voltage was swept from −2.5 V to +2.5 V and vice versa (Reproduced with permission from [22], published by published by SpringerOpen); (**c**) the measured memristance of the fabricated 3×3 memristor crossbar (Reproduced with permission from [22], published by published by SpringerOpen); (**d**) the pattern stored in the 3×3 memristor crossbar; (**e**) conceptual diagram of the single crossbar architecture for recognizing three patterns (Reproduced with permission from [16], published by IEEE).

The simulation results showed that the single crossbar array well tolerated input noise and memristance variation, in addition to saving area and power consumption. In the last part of this work, we carried out an experiment to study the performance of the single crossbar array for pattern recognition. The performance of the single crossbar array was tested on a fabricated 3×3 memristor crossbar in which each crossing point was formed by a single memristor made of carbon fiber and aluminum film, as shown in Figure 8a [22]. The carbon fiber was placed on top of the thermally evaporated aluminum film as in a stripe pattern. The fabrication process was as follows. First, aluminum (Al) wire with 100 nm thickness was evaporated on a glass substrate with a 1 mm thickness. Then, a carbon fiber with 5–10 µm diameter was placed on the patterned aluminum film. The carbon fiber and aluminum film acted as the top and bottom electrodes, respectively [23]. Figure 8b shows the switching behavior of the fabricated memristor, where the applied voltage was swept from −2.5 to 2.5 V and vice versa. For the positive sweep, SET-to-RESET switching was found around 1.7 V, as shown in Figure 8b. For the negative sweep, RESET-to-SET switching was observed around −1.8 V. Figure 8c presents the measured memristance of the fabricated 3 × 3 memristor crossbar. The crossbar with measured memristance was used to store three patterns of [LHH], [HHL], and [HLH], as represented in Figure 8d. Figure 8e shows the conceptual diagram of the single crossbar for recognizing three patterns. The input was bipolar and was generated from the raw input and its inversion, as indicated in Equation (7). Here, the column lines $i_{0}$, $i_{1}$, and $i_{2}$ represent the similarities between the input pattern and the patterns stored in the first, second, and third columns, respectively.

**Figure 9:**
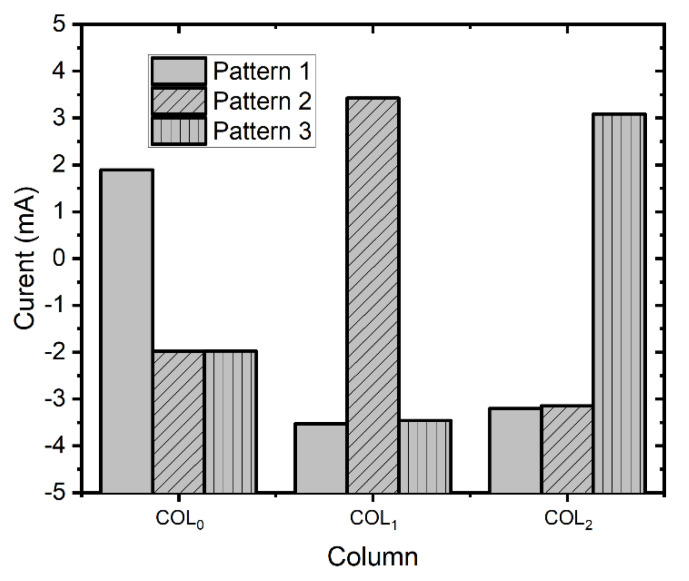
The measured column currents for the three input patterns.

To experimentally demonstrate the capability of the single crossbar array for pattern recognition, we applied the bipolar vectors obtained from patterns 1, 2, and 3 to the crossbar and measured the column currents of $i_{0}$, $i_{1}$, and $i_{2}$, respectively. Figure 9 shows the measured currents of the three columns when applying the bipolar input vectors of [HLL], [LLH], and [HLH]. 

When the bipolar vector of the [HLL] pattern was applied to the crossbar, the column current $i_{0}$ was as high as 1.9 mA, whereas the column ${\mathrm{COL}}_{1}$ and ${\mathrm{COL}}_{2}$ produced negative column currents. The obtained column current, in which $i_{0}$ was the maximum current, indicated that the first column was the best match to the input pattern. Similarly, when we applied the bipolar vector corresponding to the pattern of [HHL], the column current $i_{1}$ was as high as 3.4 mA against the negative current of $i_{0}$, and $i_{2}$, as shown in Figure 9. Moreover, the column ${\mathrm{COL}}_{2}$ had the largest current when the bipolar vector of the pattern [HLH] was applied to the crossbar. The measurement results shown in Figure 9 experimentally demonstrate that the single crossbar performed the task of pattern recognition well based on the operation of the XNOR as presented in Equation (7).

## 4. Discussion

The simulation results showed that, overall, the single crossbar architecture produced the highest recognition rate under conditions of Gaussian noise inputs and memristance variations. When input images with Gaussian noise at the SNR of −10 dB was applied to three memristor architectures, the single crossbar architecture had a recognition rate of 91%, which was 2% and 87% higher than the recognition rates of the twin crossbar and the complementary crossbar architecture, respectively. Under the condition of 40% memristance variation, the single crossbar architecture produced a recognition rate as high as 67.8%, which was 1.8% and 9.8% higher than the rates of the twin crossbar and the complementary crossbar architectures, respectively. Our experimental demonstration with a fabricated 3×3 memristor crossbar also proved the successful implementation of pattern recognition with the single crossbar architecture based on the XNOR function as presented in Equation (7).

## 5. Conclusions

A comparative study was performed on the Gaussian noise and memristance variation tolerance of the complementary crossbar architecture, the twin crossbar architecture, and the single crossbar architecture. To make the comparison, we used 10 grayscale images as input images for recognition with the three crossbar architectures. Gaussian noise was added to the input images before using the crossbar architectures for recognition. The three architectures were also tested for pattern recognition under conditions of memristance variations. The SNR value was varied from −10 to 4 dB and the percentage of memristance variation was changed from 0% to 40% to record the average recognition rates. Finally, we conducted an experiment to determine the performance of the single crossbar array architecture for pattern recognition in which a 3×3 memristor crossbar was fabricated and used for recognizing three specific patterns. Based on the simulation results, we conclude that the single crossbar architecture is the best architecture among the three architectures for image recognition under the effect of Gaussian noise and memristance variation in terms of recognition rate.
